# Upregulated Expression of ERBB2/HER2 in Multiple Myeloma as a Predictor of Poor Survival Outcomes

**DOI:** 10.3390/ijms24129943

**Published:** 2023-06-09

**Authors:** Fatih M. Uckun, Sanjive Qazi

**Affiliations:** Immuno-Oncology Program, Ares Pharmaceuticals, St. Paul, MN 55110, USA

**Keywords:** ERBB2, ERBB1, HER2, EGF, protein tyrosine kinase, multiple myeloma, transcription factor

## Abstract

The main goal of the present study was to examine if the RNA-sequencing (RNAseq)-based ERBB2/HER2 expression level in malignant plasma cells from multiple myeloma (MM) patients has clinical significance for treatment outcomes and survival. We examined the relationship between the RNAseq-based ERBB2 messenger ribonucleic acid (mRNA) levels in malignant plasma cells and survival outcomes in 787 MM patients treated on contemporary standard regimens. ERBB2 was expressed at significantly higher levels than ERBB1 as well as ERBB3 across all three stages of the disease. Upregulated expression of ERBB2 mRNA in MM cells was correlated with amplified expression of mRNAs for transcription factors (TF) that recognize the ERBB2 gene promoter sites. Patients with higher levels of ERBB2 mRNA in their malignant plasma cells experienced significantly increased cancer mortality, shorter progression-free survival, and worse overall survival than other patients. The adverse impact of high ERBB2 expression on patient survival outcomes remained significant in multivariate Cox proportional hazards models that accounted for the effects of other prognostic factors. To the best of our knowledge, this is the first demonstration of an adverse prognostic impact of high-level ERBB2 expression in MM patients. Our results encourage further evaluation of the prognostic significance of high-level ERBB2 mRNA expression and the clinical potential of ERBB2-targeting therapeutics as personalized medicines to overcome cancer drug resistance in high-risk as well as relapsed/refractory MM.

## 1. Introduction

The ERBB receptor family is comprised of four members, namely, epidermal growth factor receptor (EGFR)/ERBB1, Erb-B2 Receptor Tyrosine Kinase 2 (ERBB2/human EGFR 2 (HER2)), Erb-B2 Receptor Tyrosine Kinase 3 (ERBB3), and Erb-B2 Receptor Tyrosine Kinase 4 (ERBB4) [1,2,3,4]. ERBB1 signaling plays vital roles in regulating cell proliferation, differentiation, survival, and motility, as well as de-differentiation and malignant transformation in epithelial- or mesenchymal-lineage cells [5,6,7,8,9]. It serves as a receptor for several ligands, such as epidermal growth factor (EGF) and amphiregulin (AREG) [5,6,7,8,9]. Engagement of the ERBB1 by one of its ligands results in the activation of its catalytic protein tyrosine kinase (PTK) function and triggers tyrosine phosphorylation of related ERBB family receptors [1,2,3,4,5,6,7,8,9]. ERBB1 is overexpressed and/or intrinsically overactive due to mutations in malignant solid tumors [10,11,12,13,14]. Studies examining ERBB1 and ERBB2 overexpression and/or overactivation demonstrated significant relationships to disease progression, fast invasive growth, and metastatic spread [10,11,12,13,14], as well as resistance to biotherapeutic drugs targeting inhibitor immune checkpoints [15].

While the expression and function of the epidermal growth factor receptor (EGFR)/ERBB1 has been extensively studied in normal and malignant cells of epithelial and mesenchymal origins, very little is understood regarding its expression in normal or malignant lymphoid cells. However, recent studies indicated that lymphoid cells also express ERBB1 [16,17,18,19,20]. Notably, ERBB1 ligands AREG and EGF have been shown to stimulate the proliferation of multiple myeloma (MM) cells [21,22], and inhibiting ERBB1 caused cytotoxicity to MM cells [23,24]. Our recent studies demonstrated that ERBB1 expression is upregulated in MM cells, and higher levels of ERBB1 are associated with poor treatment outcomes and survival in newly diagnosed MM [25].

Although several ligands have been identified for ERBB1, ERBB3, and ERBB4, a specific ligand for the receptor kinase ERBB2 has not been identified [2,4]. However, ERBB2/HER2 serves as a co-receptor for other ERBB proteins that is capable of forming heterodimers and thereby facilitating the generation of an amplified intracellular biochemical signal following binding of a specific ligand to the extracellular domain of its heterodimerization partner [4]. For example, ERBB1-binding ligands, such as EGF, amphiregulin (AR), and transforming growth factor-α (TGFα) trigger a potent signal after binding to the ERBB1 portion of ERBB1×ERBB2 heterodimers [4]. The primary purpose of the present bioinformatics study was to examine if the RNA-sequencing-based ERBB2/HER2 expression level in malignant plasma cells from MM patients has clinical significance for treatment outcomes and survival. To this end, we used the Multiple Myeloma Research Foundation (MMRF)-CoMMpass RNA sequencing (RNAseq) dataset generated in patients treated on contemporary standard regimens. Upregulated expression of ERBB2 in MM cells was correlated with amplified expression of transcription factors (TF) that recognize the ERBB2 gene promoter sites. Notably, high-level ERBB2/HER2 expression was associated with increased cancer mortality and significantly worse overall survival (OS) of MM patients.

## 2. Results

### 2.1. ERBB2/HER2 mRNA Is Expressed at Significantly Higher Levels Than EGRF/ERBB1 mRNA and ERBB3 mRNA across All Clinical Stages of MM

**Figure 1:**
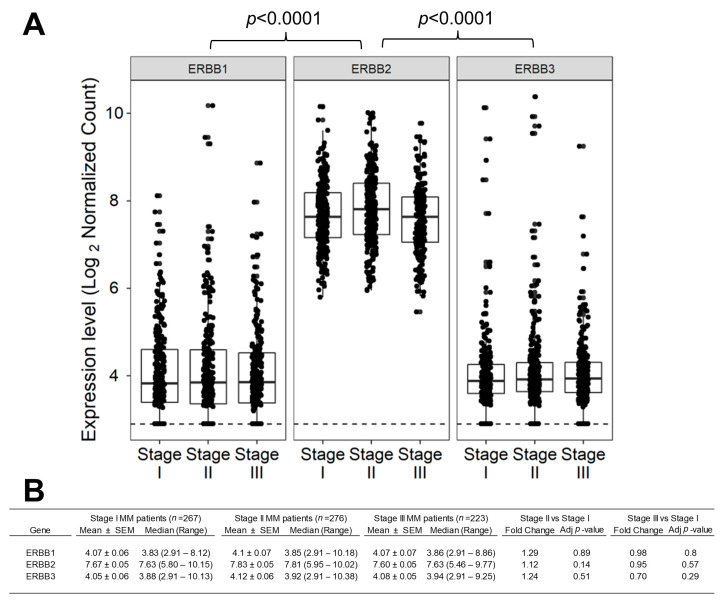
Expression of EGFR/ERBB1, ERBB2/HER2, and ERBB3 genes in malignant plasma cells from newly diagnosed MM patients. We compared the expression of the log_2_-normalized count values for ERBB1, ERBB2, and ERBB3 mRNA in malignant plasma cells from ISS stage I (*n* = 267), stage II (*n* = 276), and stage III MM patients (*n* = 223). Gene-level normalized, log_2_-transformed STAR-counts were calculated utilizing a generalized linear model that fits STAR-counts to negative binomial distribution using DESeq2_1.34.0, and a variance stabilizing data transformation was performed to account for zero count data for undetected genes using the algorithms provided in the statistical package vsn_3.62.0 implemented in R. (**A**) Depicted are the box plots representing the median expression (horizontal line in the box) in the 75th and 25th quantile box, and the whiskers representing the 3rd quartile + 1.5*(interquartile range) and 1st quartile—1.5*(interquartile range) of the expression values for ERBB1, ERBB2, and ERBB3 mRNA in stage I, stage II, and stage III MM patients. Log_2_-transformed STAR-counts are shown as log_2_-normalized counts on the Y-axis. The ERBB1 mRNA levels were recently reported [25] and they are included here solely for comparison with ERBB2 mRNA levels. We determined the lowest detection level for the normalized, variance-stabilized log_2_ count value at which there were zero alignments to the gene (detection level = 2.91 represented by the dashed line). The total number of patients with zero counts (>2.91 log_2_-transformed value) for ERBB1, ERBB2, and ERBB3 expression was 157, 0, and 62, respectively. (**B**) Pairwise comparisons for stage II versus stage I and stage III versus stage I (fold change determined from log_2_-fold difference) were carried across groups using the normalization method in the DESeq2 algorithm (implemented in R) to calculate the Wald statistic and *p*-values correcting for multiple comparisons.

The ERBB2/HER2 gene was abundantly expressed in malignant plasma cells and none of the 766 MM patients with ISS staging information had zero alignments to the ERBB2/HER2 gene (Figure 1A). No significant differences in ERBB2/HER2 expression levels were observed in the pairwise comparisons between the three stages (Figure 1B). Notably, the levels for ERBB2/HER2 mRNA in the pooled set of 766 MM patients (Mean ± SEM = 7.71 ± 0.03) was significantly higher than the levels for ERBB1 mRNA (Mean ± SEM = 4.08 ± 0.04; *t*-test, *p* < 0.0001) or ERBB3 mRNA (Mean ± SEM = 4.09 ± 0.03; *p* < 0.0001).

### 2.2. ERBB2/HER2 Expression in MM Cells Is Correlated with a Transcriptional Activator and Transcription Factors That Bind to ERBB2 Promoter Sites

**Figure 2:**
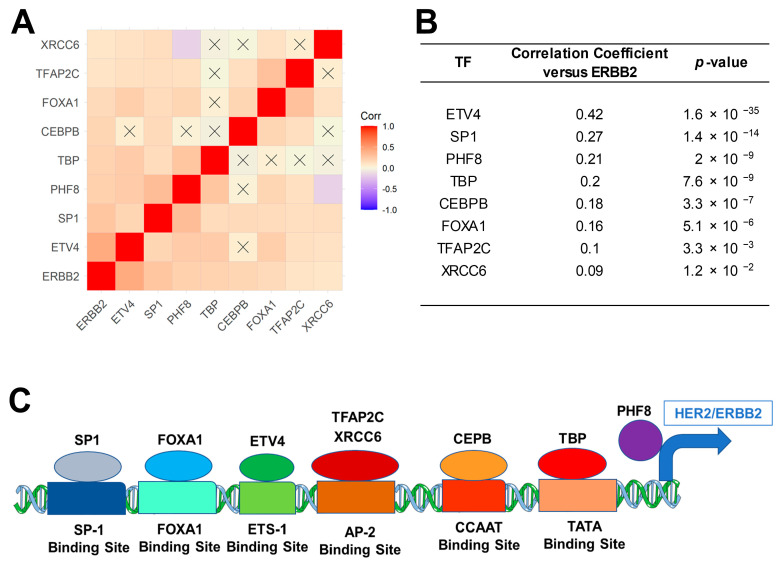
ERBB2/HER2 mRNA expression is correlated with the mRNA levels of transcription factors that bind to ERBB2 gene promoter sites. We performed pairwise Pearson correlations between mRNA levels for ERBB2 and 14 candidate TFs using the normalized count data in FPKM-UQ aligned to the reference GRCh38.p0 genome build. (**A**) Correlation coefficients for the 8 TFs that exhibited significant transcript-level correlation with ERBB2 (*p* < 0.05, FDR < 0.05; ETV4, SP1, CEBP, PHF8, TBP, FOXA1, TFAP2C, and XRCC6) were calculated across 787 MM patients (Corr) and are depicted on the heatmap ranging from positive correlations (red) to negative correlations (blue) and organized according to similarly expressed mRNAs for the TFs. Non-significant correlations are indicated with a black cross in the heat map. (**B**) Correlation coefficients are shown for 8 significantly correlated TFs, of which 5 exhibited highly statistically significant correlations with ERBB2 (*p* < 10^−6^; ETV, SP1, CEBPB, PHF8, and TBP). (**C**) Schematic representations of the transcription factor proteins (ellipses) binding to their respective DNA binding sites (rectangles), and the transcriptional activator, PHF8, are shown for the 8 most significantly positively ERBB2-correlated mRNA levels.

We correlated the mRNA expression levels of ERBB2 in malignant plasma cells from 787 MM patients with the mRNA expression levels of 14 transcription factors known to activate ERBB2 expression [26,27,28,29,30,31,32,33,34], as described in Section 2. Of these 14 genes, 8 showed statistically significant (*p* < 0.05 and FDR < 0.05) transcript-level correlation with ERBB2, namely, ETV4, SP1, CEBP, PHF8 (a transcriptional activator), TBP, FOXA1, TFAP2C, and XRCC6 (Figure 2). The biological or clinical significance of these correlations remains unknown and requires further experimental confirmation.

### 2.3. Amplified ERBB2/HER2 Expression in Malignant Plasma Cells from MM Patients Is Associated with Poor PFS Outcomes

**Figure 3:**
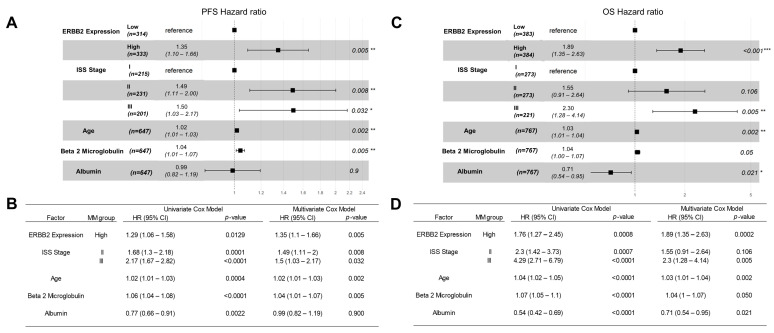
The unfavorable impact of high HER2/ERBB2 expression on PFS and OS outcomes of MM patients from the MMRF-CoMMpass study in univariate and multivariate Cox proportional hazards models. Normalized expression levels of ERRB2 (FPKM-UQ) were correlated with PFS (Panels (**A**,**B**)) and OS (Panels (**C**,**D**)) times using the univariate and multivariate Cox proportional hazards models. We investigated 2 categorical variables: (i) ERBB2 expression (i.e., ERBB2 high expression, the top 50% of patients versus ERBB2 low expression, the bottom 50% of patients) and (ii) ISS prognostic stage (ISS Stage), and 3 linear co-variates (age, serum beta 2 microglobulin levels, and serum albumin levels). Depicted in (**A**,**C**) are the forest plots along with the corresponding HRs and *p*-values for each covariate. The tables in (**B**,**D**) compare the effect of each variable considering univariate and multivariate applications of the Cox proportional hazards model. Significant *p*-values are indicated by *, **, and *** for *p* < 0.05, *p* < 0.01, and *p* < 0.001, respectively. (**A**,**B**) Comparison of the PFS times for 647 evaluable patients showed a significant increase in HR for patients with high expression of ERBB2 compared with patients with low ERBB2 expression in the multivariate model. The comparison of the multivariate model with the univariate testing of each of these variables showed that the increased HR observed in patients with high levels of ERBB2 was similar in both models: univariate model HR = 1.29 (1.06–1.58) versus multivariate model HR = 1.35 (1.1–1.66). (**C**,**D**) OS relationships were evaluable for 767 MM patients. The multivariate model exhibited an increase in HR for patients expressing high levels of ERBB2 (HR = 1.76 (1.27–2.45)) that was similar to the HR of 1.89 (1.35–2.63) in the univariate model (**D**).

We first evaluated the potential impact of high-level ERBB2/HER2 mRNA expression on PFS outcome in 669 evaluable MM patients (Figure S1A). ERBB2^high^ patients (i.e., 335 patients with the top 50% of the highest observed expression level for FPKM-UQ aligned to the ERBB2 sequence) had significantly shorter median PFS than ERBB2^low^ patients (i.e., 334 patients with the bottom 50% of the expression level for FPKM-UQ aligned to the ERBB2 sequence) (27 months vs. 36 months, log-rank chi-square = 4.85, *p*-value = 0.028). We confirmed the adverse impact of high ERBB2/HER2 expression on PFS using both univariate and multivariate Cox regression models for 647 evaluable patients (Figure 3A). Further, in the multivariate Cox regression model, ERBB2 mRNA expression level as a linear covariate exhibited a significant 1.54-fold increase in the hazard ratio for each unit increase in FPKM-UQ counts (*p* < 0.001) that persisted with the inclusion of other prognostic factors, including ISS stage, age, as well as serum albumin and beta 2 microglobulin levels (Figure S1B).

### 2.4. Amplified ERBB2/HER2 Expression in Malignant Plasma Cells from MM Patients Is Associated with Poor OS

**Figure 4:**
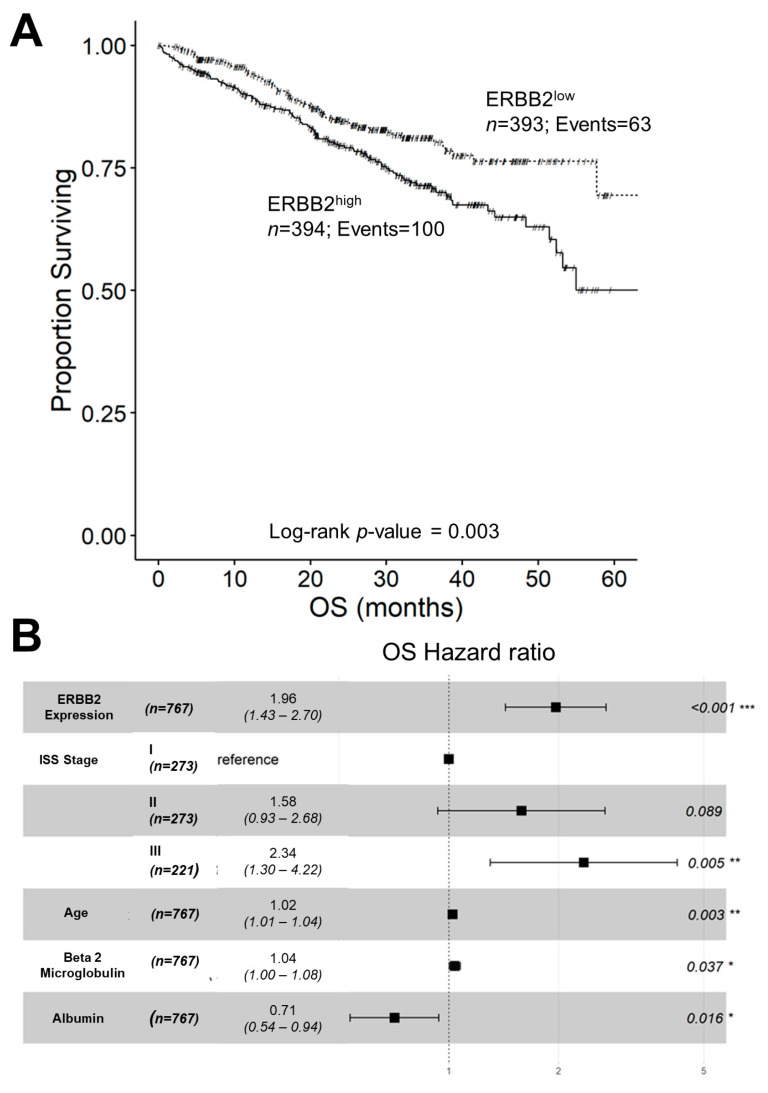
Higher expression levels of HER2/ERBB2 in malignant plasma cells from MM patients are associated with shorter OS. (**A**) Depicted for comparison are the OS curves for 394 ERBB2^high^ MM patients (i.e., patients with the top 50% of the highest observed expression level for FPKM-UQ aligned to the ERBB2 sequence) and 393 ERBB2^low^ MM patients (i.e., patients with the bottom 50% with the lowest observed expression level for FPKM-UQ aligned to the ERBB2 sequence). Cumulative proportions surviving at 60 mo: 50.1 ± 6.7% for ERBB2^high^ and 69.5 ± 7.2% for ERBB2^low^. (**B**) Depicted is the forest plot comparing the HRs for each covariate included in a multivariate Cox proportional hazards model, namely, ERBB2 mRNA level, ISS stage, age, as well as serum albumin and beta 2 microglobulin levels in 767 evaluable patients. ERBB2 mRNA expression level exhibited a significant 1.96-fold increase in the hazard ratio for each unit increase in FPKM-UQ counts (*p* < 0.001). Significant *p*-values are indicated by *, **, and *** for *p* < 0.05, *p* < 0.01, and *p* < 0.001, respectively.

We next sought to determine the effect of amplified ERBB2/HER2 mRNA expression on OS in 787 MM patients considering all deaths from any cause in our analysis. ERBB2^high^ patients (i.e., 394 patients with the top 50% of the highest observed expression level for FPKM-UQ aligned to the ERBB2 sequence) had a significantly shorter OS than ERBB2^low^ patients (i.e., 393 patients with the bottom 50% of the observed expression level for FPKM-UQ aligned to the ERBB2 sequence) had significantly worse OS outcomes. The cumulative proportion of ERBB2^high^ patients remaining alive at 60 months was 50.1 ± 6.7%, whereas the cumulative proportion of ERBB2^low^ patients remaining alive at 60 months was 69.5 ± 7.2% (log-rank chi-square = 8.82, *p*-value = 0.003) (Figure 4A). We confirmed the adverse impact of high ERBB2 expression on OS using both univariate and multivariate Cox regression models for 767 evaluable patients (Figure 3B). Further, in the multivariate Cox regression model, ERBB2 mRNA expression level as a linear covariate exhibited a significant two-fold increase in the hazard ratio for each unit increase in FPKM-UQ counts (*p* < 0.001) that persisted with the inclusion of other prognostic factors, including ISS stage, age, as well as serum albumin and beta 2 microglobulin levels (Figure 4B).

We next assessed the OS of four distinct patient groups defined by co-expression levels of ERBB2 and ERBB1: (i) ERBB1^high^/ERBB2^high^ (top 50th percentile of the highest observed expression levels for both ERBB1 and ERBB2 mRNA expression levels) (*n* = 195), (ii) ERBB1^low^/ERBB2^low^ (bottom 50th percentile of the lowest observed expression levels for both ERBB1 and ERBB2 (*n* = 194), (iii) ERBB1^low^/ERBB2^high^ (*n* = 199), and (iv) ERBB1^high^/ERBB2^low^(*n* = 199). ERBB1^low^/ERBB2^low^ patients representing 24.7% of the 787 evaluable patients had significantly fewer deaths and consequently the best 5-year survival of 83.3% when compared to the remaining three groups of patients (Figure S2).

### 2.5. Amplified ERBB2/HER2 Expression in Malignant Plasma Cells from MM Patients Is Associated with Increased Cancer-Related Mortality

We next evaluated the potential impact of high-level ERBB2/HER2 mRNA expression on cancer-related mortality (CRM) in 716 evaluable MM patients (Figure S3A). ERBB2^high^ patients (i.e., 358 patients with the top 50% of the observed expression level for FPKM-UQ aligned to the ERBB2 sequence) had a significantly higher CRM than ERBB2^low^ patients (i.e., 358 patients with the bottom 50% of the observed expression level for FPKM-UQ aligned to the ERBB2 sequence) (37.3 ± 7.2% vs. 22.7 ± 8.5%; log-rank chi-square = 6.17, *p*-value = 0.013). We confirmed the adverse impact of high ERBB2 expression on CRM using a multivariate Cox regression model for 695 evaluable patients. ERBB2 mRNA expression level as a linear covariate exhibited a significant 2.23-fold increase in the hazard ratio for each unit increase in FPKM-UQ counts (*p* < 0.001) that persisted with the inclusion of other prognostic factors, including ISS stage, age, as well as serum albumin and beta 2 microglobulin levels (Figure S3B).

We next assessed the CRM of four distinct patient groups defined by co-expression levels of ERBB2 and ERBB1: (i) ERBB1^high^/ERBB2^high^ (the top 50% of the observed expression levels for both ERBB1 and ERBB2 mRNA expression levels) (*n* = 181), (ii) ERBB1^low^/ERBB2^low^ (the bottom 50% of the observed expression levels for both ERBB1 and ERBB2 (*n* = 181), (iii) ERBB1^low^/ERBB2^high^ (*n* = 177), and (iv) ERBB1^high^/ERBB2^low^(*n* = 177). ERBB1^low^/ERBB2^low^ patients representing 25.3% of the 716 evaluable patients had significantly fewer cancer-related deaths and, consequently, the lowest 5-year CRM (7.2 ± 2.1%) when compared with the remaining three groups of patients (5-year CRM: ERBB1^high^/ERBB2^high^ = 39.4 ± 10%, ERBB1^high^/ERBB2^low^ = 38.1 ± 14.4%, and ERBB1^low^/ERBB2^high^ = 33.8 ± 9%) (Figure S4).

### 2.6. A STRING Model of HER2/ERBB2-Regulated Signaling Network Reveals 8 ERBB2-Associated Signaling Molecules as Poor OS Indicators

**Figure 5:**
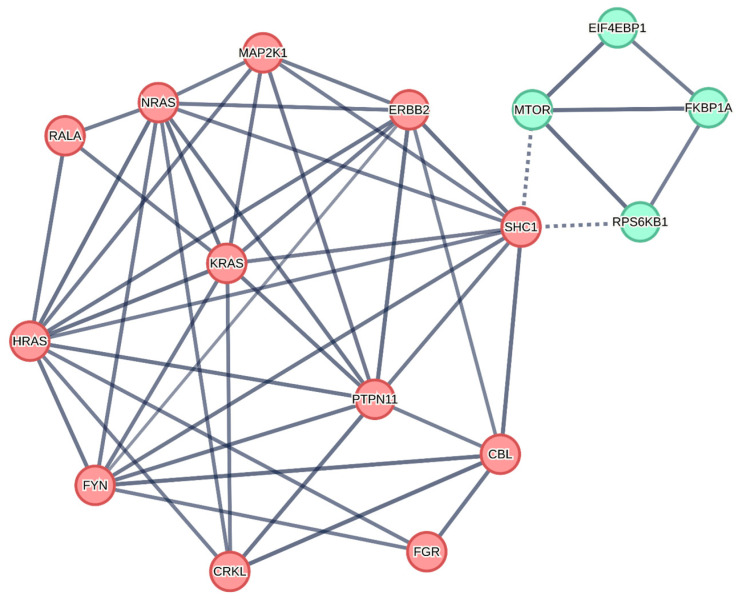
Higher expression levels of HER2/ERBB2 and a highly connected network of genes coding for signaling proteins in malignant plasma cells from MM patients are associated with worse OS outcomes. Depicted is a model STRING network comprised of ERBB2 and 15 other signaling molecules with poor survival associated with their high-level mRNA expression. The networked associations were calculated using the strength of evidence from experiments, databases, and co-expression using the STRING algorithm (STRING11.5 algorithm (http://string-db.org/). Clustering was conducted on the association scores to group protein–protein interaction networks using the MCL algorithm provided via the STRING software showing solid lines representing connections within the 2 detected clusters and dotted lines connecting between clusters. The network graph illustrates 16 nodes with 49 edges which were significantly higher than the expected number of 8 edges (*p* < 0.0001) based on a random set of proteins of the same size and degree of distribution drawn from the genome, suggesting that this highly connected set of nodes is biologically meaningful. In this network graph, ERBB2 exhibited direct associations with 8 genes coding for proteins, namely, SHC1, CBL, PTPN11, FYN, KRAS, HRAS, NRAS, and MAP2K1, and an additional 7 genes with associations in the second shell of this network: EIF4EBP1; MTOR; FGR; FKBP1A; RALA; CRKL; and RPS6KB1.

We compared the OS outcomes curves for 394 MM patients with high levels of mRNA expression for ERBB2 and associated genes coding for proteins associated with ERBB2 (i.e., patients with the top 50% of the observed expression level for FPKM-UQ values for each gene) with the OS outcomes of 393 MM patients with low mRNA expression levels for each gene (i.e., patients with the bottom 50% of the observed expression level for FPKM-UQ values). Depicted in Figure 5 are ERBB2 and 15 additional genes in the STRING network exhibiting prognostic significance. ERBB2 exhibited direct associations with SHC1, CBL, PTPN11, FYN, KRAS, HRAS, NRAS, and MAP2K1 (Figure 5). Three signaling molecules exhibited the most significant increases in hazard ratios at augmented expression levels, namely, EIF4EBP1 (HR = 1.84; *p* = 0.0001), PTPN11 (HR = 1.81; *p* = 0.0002), and MAP2K1 (HR = 1.67; *p* = 0.0011) (Table 1).

## 3. Discussion

Contemporary treatment strategies for multiple myeloma (MM) still fail in a significant portion of patients due to the emergence of resistance in MM clones to active anti-MM drugs [35,36,37,38,39,40,41]. Effective treatments capable of preventing or overcoming cancer drug resistance are urgently needed [35,36,37,38,39,40,41]. The tumor microenvironment (TME) in MM has been shown to play a pivotal role in disease progression by facilitating the immune escape of MM cells via both immune suppression and activation of signaling pathways that prevent apoptosis and stimulate their proliferation [42,43,44,45,46,47,48]. The immunosuppressive cellular elements of the TME in MM include regulatory T cells (Tregs), regulatory B cells (Bregs), and myeloid-derived suppressor cells (MDSC), whereas the humoral elements include several growth factors and cytokines such as IL-10 that activates Tregs and M2 macrophages and TGF-β which inhibits cytotoxic T-cells and NK cells [42,43,44,45,46,47,48,49]. Several cytokines and growth factors are abundantly produced in the TME of MM patients, including the ERBB1 ligands EGF, TGF-α, and AR, and cause activation of signal transduction pathways that have been implicated in the proliferation, prolonged survival, and dissemination of MM cells [50,51,52,53,54,55,56]. New therapeutic strategies targeting the TME in MM are being explored in preclinical and clinical settings [42,43,44,45,46,47,48,49].

ERBB2/HER2 serves as a co-receptor for ERBB1 and as a heterodimer with ERBB1 contributing to the generation of an amplified intracellular biochemical signal after binding of an ERBB1 ligand to the ERBB1 portion of the ERBB1×ERBB2 heterodimer [4]. Upregulated ERBB2/HER2 expression could contribute to the formation of ERBB1×ERBB2 heterodimers and thereby potentiate the ability of ERBB1 ligands in the MM TME, such as EGF and AR to promote the survival, proliferation, and dissemination of MM cells. Patients with higher levels of ERBB2 mRNA in their malignant plasma cells experienced significantly increased CRM, shorter PFS, and worse OS than other patients. The adverse impact of high ERBB2/HER2 expression on patient survival outcomes remained significant in multivariate Cox proportional hazards models that accounted for the effects of other prognostic factors.

**Figure 6:**
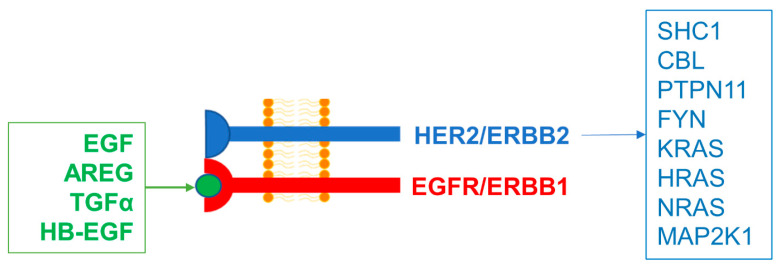
Amplified HER2/ERBB2 expression and directly associated signaling molecules identified via the STRING algorithm resulting in poor overall survival in MM patients. The HER2/ERBB2 receptor (blue symbol) does not bind to the ligands but dimerizes with the EGFR/ERBB1 receptor (red symbol) to activate downstream signal transduction pathways. Dimers of EGFR/ERBB1 bind to EGFR/ERBB1 ligands (green circle; a select group of ligands shown in the green box including EGF, amphiregulin (AREG), transforming growth factor-alpha (TGFα), and heparin-binding EGF-like growth factor (HB-EGF)) to send signals into the cell. Depicted are the 8 signaling molecules directly associated with ERBB2 from the proposed interaction network identified via the STRING algorithm that also exhibited poor OS outcomes at high levels of mRNA expression.

In MM cells with low-level ERBB1 expression unlikely to form ERBB1xERBB1 homodimers abundantly, abundant expression of ERBB2 could compensate for the low-level ERBB1 expression by enabling the formation of ERBB1×ERBB2 heterodimers. It is noteworthy that the CRM of ERBB1^low^ERBB2^high^ patients was as high as the CRM of ERBB1^high^ patients with low or high ERBB2 levels (Figure S4). We propose a model according to which ERBB1 signals transmitted by activated ERBB1×ERBB1 homodimers or ERBB1×ERBB2 heterodimers contribute to the demonstrated adverse prognostic effects of ERBB1 [25] and ERBB2/HER2 (present study) in MM (Figure 6). ERBB2 exhibited direct links with eight signaling molecules that were associated with significant increases in hazard ratios for poor OS (Table 1, Figure 5). In agreement with such a model, ERBB1^lowand^ ERBB2^low^ patients whose MM cells would be less likely to form biologically meaningful quantities or ERBB1xERBB1 homodimers or ERBB1×ERBB2 heterodimers had the lowest 5-year cancer-related mortality (7.2 ± 2.1%) when compared with the remaining patients (Figure S4).

Several ERBB2/HER-2 targeting therapeutics have been approved for the treatment of solid tumors, such as breast cancer and lung cancer [57,58,59]. Targeting ERBB2 with FDA-approved small molecule ERBB2 inhibitors as well as monoclonal antibodies could potentially improve the treatment options for high-risk or relapsed/refractory (R/R) MM patients. Our results presented herein warrant further examination of the therapeutic potential of already FDA-approved small molecule inhibitors as well as monoclonal antibodies targeting ERBB2 that could be repurposed for use in high-risk or R/R MM. The evaluation of the clinical potential of ERBB2/HER-2 targeting therapeutics, including monoclonal antibodies trastuzumab and pertuzumab, antibody-drug conjugates trastuzumab emtansine and trastuzumab deruxtecan, and small molecule TKI tucatinib alone and in combination with standard anti-MM drugs, as well as ERBB1-targeting antibodies and TKI, would seem warranted. The dual function of TKI neratinib and palatinib inhibit both ERBB1 and ERBB2 and may have clinical potential for high-risk or R/R MM patients.

In this study, we demonstrated that ERBB2/HER2 co-receptor mRNA was expressed in malignant plasma cells from MM patients at significantly higher levels than ERBB1 as well as ERBB3 across all three stages of the disease. We tested the hypothesis that the amplified ERBB2 mRNA levels in MM cells may be driven by increased expression of specific TFs that transcriptionally activate ERBB2 expression. Our findings demonstrated for the first time that increased ERBB2 mRNA expression shows statistically significant correlations with upregulated mRNA levels of several such TFs, including ETV, SP1, CEBPB, PHF8, and TBP. We propose a model according to which the observed upregulation of ERB2/HER2 mRNA expression in MM cells is driven transcriptionally by several TFs (Figure 2C). The presented data expand our current knowledge regarding the networks of signaling pathways affecting the biology and clinical outcome of MM as well as emerging new molecular targets for chemotherapy-drug-resistant MM [60,61,62,63,64,65,66,67,68,69].

Our hypothesis-generating study suffers from a number of limitations including its primary focus on bioinformatics-based analyses without integrated laboratory testing of ERBB2 mRNA levels using other methods in addition to RNAseq and lack of data to show that MM cells from ERBB2^high^ patients with high levels of ERBB2 mRNA also express ERBB2 protein at higher levels than ERBB2^low^ patients. Our results need to be verified in a prospective hypothesis-testing study with integrated RNAseq and biochemical testing in a large MM patient population. A multivariate analysis of the prognostic significance of ERBB2 overexpression in relationship to other prognostic factors and especially patient treatment will be very important.

## 4. Materials and Methods

### 4.1. Processing and Analysis of the Multiple Myeloma Research Foundation (MMRF)-CoMMpass RNA Sequencing (RNAseq) Dataset

The processing of the MMRF-CoMMpass RNAseq dataset was performed, as previously detailed [25]. Pre-processed RNAseq data files for 787 cases used in our analysis are accessible via the GDC portal (https://gdc.cancer.gov/about-gdc/contributed-genomic-data-cancer-research/foundation-medicine/multiple-myeloma-research-foundation-mmrf, accessed on 20 March 2023). The analytical path for RNAseq datasets employed standardized mRNA quantification techniques to enable meta-analysis across multiple projects, as previously described in detail [25]. We utilized the latest version of the RNAseq STAR-quantified data for all 787 cases performed on the GDC portal (https://gdc.cancer.gov/about-gdc/contributed-genomic-data-cancer-research/foundation-medicine/multiple-myeloma-research-foundation-mmrf, accessed on 20 March 2023, release date: 29 March 2022, version 32). STAR-aligned read groups were quantified using the two-pass method that generated the final alignments onto the GRCh38.p0 reference genome to calculate the gene-level RNAseq raw STAR-count data (i.e., STAR-aligned unstranded number of reads aligned per gene per sample), and fragments per kilobase of transcript per million mapped reads (FPKM) normalized to the upper quartile FPKM (FPKM-UQ). Gene expression profiles were downloaded from the archived MMRF CoMMpass dataset, as previously reported [25].

Patient-level clinical data were processed via functional tools provided in GenomicDataCommons_1.18.0 and the case IDs were matched with RNAseq unique identifiers utilizing the metadata (“metadata.cart.2022-09-04.json”) from the GDC portal converted into R data by running the utilities rjson_0.2.21 and stringr_1.4.0. The database included 766 ISS-staged patients, including 267 stage I, 276 stage II, and 223 stage III patients. We compared the mRNA expression levels for EGFR/ERBB1, ERBB2/HER2, and ERBB3 in each subset using the DESeq2 package (DESeq2_1.34.0 implemented using R version 4.1.2; R Foundation for Statistical Computing, Vienna, Austria. (1 November 2021)) [70]. Gene-level normalized, log_2_-transformed STAR-counts were calculated utilizing a generalized linear model that fits STAR-counts to negative binomial distribution to approximate the empirical distribution of the count data [25,70]. Statistical significance was assessed by testing the null hypothesis that there is no differential expression across the two sample groups (Log_2_ fold change = 0) using the Wald test [25,70] reporting the test statistic and *p*-value for each gene. Genes were considered differentially expressed if the *p*-values were less than 0.05 after adjusting for multiple comparisons using the Benjamini and Hochberg method [25,70,71]. To visualize the gene expression levels in box plots, the normalized log_2_ values were calculated from the RNAseq count data using the variance stabilization method supplied by the algorithms in vsn_3.62.0 [72]. Pairwise t-tests were performed to compare the expression of ERBB1 vs. ERBB2 and ERBB3 vs. ERBB2 across 766 patients and corrected for multiple comparisons using the Benjamini and Hochberg method.

We correlated the mRNA expression levels of ERBB2/HER2 in malignant plasma cells from 787 MM patients with the mRNA expression levels of 14 transcription factors known to activate ERBB2 expression [26,27,28,29,30,31,32,33,34] represented by ETS Variant Transcription Factor 4 (ETV4), Sp1 Transcription Factor (SP1), CCAAT Enhancer Binding Protein Beta (CEBPB), TATA-Box Binding Protein (TBP), Forkhead Box A1 (FOXA1), Transcription Factor AP-2 Gamma (TFAP2C), X-Ray Repair Cross Complementing 6 (XRCC6), CCAAT Enhancer Binding Protein Alpha (CEBPA), X-Ray Repair Cross Complementing 5 (XRCC5), E74 Like ETS Transcription Factor 3 (ELF3), Transcription Factor AP-2 Beta (TFAP2A), YY1 Transcription Factor (YY1) and ETS Proto-Oncogene 1, Transcription Factor (ETS1), and a transcription activator (PHF8).

Pairwise correlation coefficients were determined for 240 gene combinations using the FPKM-UQ data for each gene. Correlation coefficients were visualized on a heatmap color coded for positive correlations (red = +1) to negative correlations (blue = −1). The clustering algorithm identified co-regulated sets of mRNA expression values using the statistical package ggcorrplot_0.1.3 implemented in R software (R Foundation for Statistical Computing, Vienna, Austria). Significant correlations were identified for *t*-test *p*-values less than 0.05 and false discovery rates (FDRs) less than 0.05.

### 4.2. Analysis of Patient Outcomes According to ERBB2/HER2 mRNA Expression Levels

RNAseq and overall survival (OS) data were available for 787 MM patients. Progression-free survival (PFS) information was available for 669 MM patients. The Kaplan–Meier (KM) method was employed utilizing the software packages survival_3.2-13, survminer_0.4.9, and survMisc_0.5.5 operated in the R environment to compare the progression-free survival (PFS) (*n* = 669), overall survival (OS) (*n* = 787), and cancer-related mortality (CRM) (*n* = 716) outcomes of patient subsets, as previously described [25]. Log-rank *p*-values less than 0.05 were deemed significant. Graphical representations of the survival curves were visualized using graph-drawing packages implemented in the R programming environment: dplyr_1.0.7, ggplot2_3.3.5, and ggthemes_4.2.4. The percentiles of patients expressing ERBB2/HER2 were determined using the metric fragments per kilobase of transcript per million mapped reads (FPKM) calculation normalized to the upper quartile FPKM (FPKM-UQ) for the whole geneset in each sample. PFS, OS, and cancer-related mortality (CRM) curves were compared for patients expressing high levels of ERBB2 (top 50th percentile of FPKM-UQ values) versus low levels of ERBB2 (bottom 50th percentile of FPKM-UQ values). Univariate Cox regression analyses were also performed to evaluate the impact of high-level ERBB2 expression on PFS and OS outcomes, as previously described [25]. We also investigated cancer-related mortality of four groups of patients with combinations of expression levels of ERBB1 and ERBB2: patients expressing high levels of both ERBB1 and ERBB2 (ERBB1^high^/ERBB2^high^; top 50th percentile for both ERBB1 and ERBB2 mRNA expression); low levels of ERBB1 and ERBB2 (ERBB1^low^/ERBB2^low^; below the 50th percentile of the lowest observed expression levels for both ERBB1 and ERBB2); and crossed combinations of ERBB1 and ERBB2 mRNA expression levels (ERBB1^low^/ERBB2^high^, and ERBB1^high^/ERBB2^low^).

Multivariate analyses of the potential effect of the MMRF CoMMpass ERBB2/HER2 cohort groups on the PFS, OS, and cancer-related mortality outcomes were performed using the multivariate Cox proportional hazards model to adjust for other patient risk factors and staging criteria, as previously described [25]. Briefly, the model included (i) the mRNA expression level for ERBB2 as a linear co-variate or as a categorical variable comparing high versus low ERBB2 mRNA expression levels (50% cut-off for the range of FPKM-UQ values), (ii) the prognostic staging data according to the International Staging System (ISS), (iii) age, (iv) serum concentrations for albumin concentration and beta 2 microglobulin level, implemented in R (survival_3.2-13 ran in R version 4.1.2. Forest Plots were utilized, as previously reported [25]), to visualize the hazard ratios for Cox proportional hazards models for PFS, OS, and cancer-related mortality (survminer_0.4.9 ran in R version 4.1.2 (1 November 2021)). The life table hazard ratios (HRs) were estimated using the exponentiated regression coefficient for Cox proportional hazards analyses implemented in R (survival_3.2-13 ran in R version 4.1.2.).

### 4.3. Identifying Prognostically Relevant Signaling Proteins Networked to ERBB2 Expression Using the STRING Interaction Algorithm

Putative protein–protein interaction networks derived from mRNA levels with prognostic impact on the OS were constructed using the STRING11.5 algorithm (http://string-db.org/, accessed 20 May 2023) to identify candidate hub proteins connecting ERBB2 gene expression to potential networked interactions involving intracellular signaling molecules [71]. In these diagrams, the nodes depicted protein identifiers, and the edges depicted associations between the proteins.

Networks were visualized by first seeding the inputs and then growing the networks to identify connecting hubs between the inputs. The edges indicated the confidence level for the association calculated from various lines of experimental evidence (experiments, databases, and co-expression). Interaction scores greater than 0.7 were considered to define associations between the proteins and indicated by the thickness of the connecting lines for scores of 0.7 and 0.9. In the first instance, ERBB2 was seeded with the genes whose mRNA expression level was equal to or greater than the 50th percentile resulting in worse overall survival outcomes in 787 MM patients, namely, SHC1, CBL, PTPN11, FYN, KRAS, HRAS, NRAS, and MAP2K1.

These genes served as inputs to identify additional interactants by interrogating the STRING database for associations with scores equal to or greater than 0.7, no more than 20 interactants in the first shell, and no more than 5 interactants in the second shell of networked proteins. This iterative process identified a full module of 16 genes including ERBB2 whose augmented expression was highly associated with poor OS outcomes (*p* < 0.05, FDR < 0.1). A Kaplan–Meier overall survival analysis was performed to compare the top 50th percentile expression of each gene coding for the networked protein versus the bottom 50th percentile expression that resulted in 16 genes exhibiting worse survival outcomes (Log-rank *p*-values < 0.05, FDR = 0.06) at higher levels of expression in MM patients, namely: CBL, CRKL, EIF4EBP1, ERBB2, FGR, FKBP1A, FYN, HRAS, KRAS, MAP2K1, MTOR, NRAS, PTPN11, RALA, RPS6KB1, and SHC1. A Markov cluster (MCL) algorithm was applied to visualize subnetworks of highly connected nodes in the 16 genes coding for proteins shown to result in worse survival outcomes in MM patients. The granularity of visualizing the subnetworks is controlled via the Markov cluster inflation factor ranging from 1.1 to 10 where, in our analysis, we discerned 2 highly connected sub-networks by setting the value to 2.1 for the 16 genes examined.

## 5. Conclusions

In MM patients, ERBB2 was expressed at significantly higher levels than ERBB1 as well as ERBB3 across all three stages of the disease. Upregulated expression of ERBB2 mRNA in MM cells was correlated with amplified expression of mRNAs for transcription factors that recognize the ERBB2 gene promoter sites. Patients with higher levels of ERBB2 mRNA in their malignant plasma cells experienced significantly increased cancer mortality, shorter progression-free survival, and worse overall survival than other patients. The adverse impact of high ERBB2 expression on patient survival outcomes remained significant in multivariate Cox proportional hazards models that accounted for the effects of other prognostic factors.

## Figures and Tables

**Table 1:** ERBB2-associated signaling molecules as poor OS indicators.

Signaling Molecule	Hazard Ratio (95% CI)	Log-Rank Chi-Square Value	*p*-Value
EIF4EBP1	1.84 (1.34–2.53)	14.42	0.0001
PTPN11	1.81 (1.31–2.49)	13.58	0.0002
MAP2K1	1.67 (1.23–2.29)	10.71	0.0011
ERBB2	1.61 (1.17–2.2)	8.82	0.0030
FYN	1.58 (1.16–2.17)	8.35	0.0039
MTOR	1.56 (1.13–2.14)	7.64	0.0057
SHC1	1.54 (1.13–2.11)	7.43	0.0064
KRAS	1.53 (1.12–2.08)	7.36	0.0067
FGR	1.48 (1.09–2.01)	6.21	0.0127
FKBP1A	1.49 (1.08–2.04)	6.15	0.0132
NRAS	1.46 (1.07–1.99)	5.83	0.0158
HRAS	1.46 (1.07–1.99)	5.74	0.0166
CBL	1.46 (1.06–2)	5.55	0.0185
RALA	1.42 (1.04–1.94)	4.90	0.0269
CRKL	1.38 (1.01–1.88)	4.13	0.0422
RPS6KB1	1.36 (1–1.86)	3.94	0.0471

EIF4EBP1: eukaryotic translation initiation factor 4E-binding protein 1; PTPN11: tyrosine-protein phosphatase non-receptor type 11; MAP2K1: dual specificity mitogen-activated protein kinase kinase 1/MEK1; ERBB2: receptor tyrosine-protein kinase erbB-2; FYN: tyrosine-protein kinase Fyn; MTOR: serine/threonine-protein kinase mTOR; SHC1: SHC-transforming protein 1; KRAS: GTPase KRas; FGR: Fgr proto-oncogene, Src family tyrosine kinase; FKBP1A: peptidyl-prolyl cis–trans isomerase; NRAS: Nras proto-oncogene, gtpase; HRAS: GTPase HRas; CBL: E3 ubiquitin-protein ligase CBL; RALA: Ras-related protein Ral-A; CRKL: Crk-like protein; and RPS6KB1: ribosomal protein S6 kinase beta-1.

## Data Availability

All data are presented in the manuscript. The publicly available archived databases that were used to generate the data are provided in Section 2. Gene level RNAseq raw count data (STAR-aligned unstranded number of reads aligned per gene per sample) were downloaded from the archived MMRF CoMMpass dataset by connecting to the GDC portal (https://gdc.cancer.gov/about-gdc/contributed-genomic-data-cancer-research/foundation-medicine/multiple-myeloma-research-foundation-mmrf (accessed on 24 September 2022)) using the Bioconductor package, GenomicDataCommons_1.18.0 (release date 11 August 2021 with full functionality as provided by TCGAbiolinks for accessing GDC data (https://bioconductor.org/packages/release/bioc/html/GenomicDataCommons.html (accessed on 20 September 2022)) implemented in R version 4.1.2 (1 November 2021). The mRNA expression data were deposited in files appended with “….rna_seq.augmented_star_gene_counts.tsv”. Clinical data for each MM patient were also acquired via functions provided in GenomicDataCommons_1.18.0 and the case IDs were matched with RNAseq unique identifiers utilizing the metadata (“metadata.cart.2022-09-04.json”) from the GDC portal converted into R data by running the utilities rjson_0.2.21 and stringr_1.4.0.

## Supplementary Materials

The supporting information can be downloaded at: https://www.mdpi.com/article/10.3390/ijms24129943/s1.

## References

[1] Olayioye M.A., Neve R.M., Lane H.A., Hynes N.E. (2000). The ErbB signaling network: Receptor heterodimerization in development and cancer. EMBO J..

[2] Jones J.Y., Akita R.W., Sliwkowski M. (1999). Binding specificities and affinities of egf domains for ErbB receptors. FEBS Lett..

[3] Hassan G., Seno M. (2022). ERBB Signaling Pathway in Cancer Stem Cells. Adv. Exp. Med. Biol..

[4] Arkhipov A., Shan Y., Kim E.T., O Dror R., E Shaw D. (2013). Her2 activation mechanism reflects evolutionary preservation of asymmetric ectodomain dimers in the human EGFR family. eLife.

[5] Wells A. (1999). EGF receptor. Int. J. Biochem. Cell Biol..

[6] Sabbah D.A., Hajjo R., Sweidan K. (2020). Review on Epidermal Growth Factor Receptor (EGFR) Structure, Signaling Pathways, Interactions, and Recent Updates of EGFR Inhibitors. Curr. Top. Med. Chem..

[7] Wang Z. (2017). ErbB Receptors and Cancer. Methods Mol. Biol..

[8] Santos E.D.S., Nogueira K.A.B., Fernandes L.C.C., Martins J.R.P., Reis A.V.F., Neto J.D.B.V., Júnior I.J.D.S., Pessoa C., Petrilli R., Eloy J.O. (2021). EGFR targeting for cancer therapy: Pharmacology and immunoconjugates with drugs and nanoparticles. Int. J. Pharm..

[9] Hynes N.E., Lane H.A. (2005). ERBB receptors and cancer: The complexity of targeted inhibitors. Nat. Rev. Cancer.

[10] Ho C., Laskin J. (2009). EGFR-directed therapies to treat non-small-cell lung cancer. Expert Opin. Investig. Drugs.

[11] Ciardiello F., Tortora G. (2008). EGFR Antagonists in Cancer Treatment. N. Engl. J. Med..

[12] Marquez-Medina D., Popat S. (2015). Afatinib: A second-generation EGF receptor and ErbB tyrosine kinase inhibitor for the treatment of advanced non-small-cell lung cancer. Futur. Oncol..

[13] Singh D., Attri B.K., Gill R.K., Bariwal J. (2016). Review on EGFR Inhibitors: Critical Updates. Mini-Rev. Med. Chem..

[14] Cai W.-Q., Zeng L.-S., Wang L.-F., Wang Y.-Y., Cheng J.-T., Zhang Y., Han Z.-W., Zhou Y., Huang S.-L., Wang X.-W. (2020). The Latest Battles Between EGFR Monoclonal Antibodies and Resistant Tumor Cells. Front. Oncol..

[15] Kumagai S., Koyama S., Nishikawa H. (2021). Antitumour immunity regulated by aberrant ERBB family signalling. Nat. Rev. Cancer.

[16] Zaiss D.M.W., van Loosdregt J., Gorlani A., Bekker C.P.J., Gröne A., Sibilia M., van Bergen en Henegouwen P.M.P., Roovers R.C., Coffer P.J., Sijts A.J.A.M. (2013). Amphiregulin Enhances Regulatory T Cell-Suppressive Function via the Epidermal Growth Factor Receptor. Immunity.

[17] Minutti C.M., Drube S., Blair N., Schwartz C., McCrae J.C., McKenzie A.N., Kamradt T., Mokry M., Coffer P.J., Sibilia M. (2017). Epidermal Growth Factor Receptor Expression Licenses Type-2 Helper T Cells to Function in a T Cell Receptor-Independent Fashion. Immunity.

[18] Zeboudj L., Maître M., Guyonnet L., Laurans L., Joffre J., Lemarie J., Bourcier S., Nour-Eldine W., Guérin C., Friard J. (2018). Selective EGF-Receptor Inhibition in CD4+ T Cells Induces Anergy and Limits Atherosclerosis. J. Am. Coll. Cardiol..

[19] Wang L., Huang Z., Huang W., Chen X., Shan P., Zhong P., Khan Z., Wang J., Fang Q., Liang G. (2017). Inhibition of epidermal growth factor receptor attenuates atherosclerosis via decreasing inflammation and oxidative stress. Sci. Rep..

[20] Spengeman J.D., Green T.D., Bertrand F.E., McCubrey J.A. (2005). Activated EGFR Promotes the Survival of B-Lineage Acute Leukemia in the Absence of Stromal Cells. Cell Cycle.

[21] Mahtouk K., Hose D., Rème T., De Vos J., Jourdan M., Moreaux J., Fiol G., Raab M., Jourdan E., Grau V. (2005). Expression of EGF-family receptors and amphiregulin in multiple myeloma. Amphiregulin is a growth factor for myeloma cells. Oncogene.

[22] Luo H., Zhang D., Wang F., Wang Q., Wu Y., Gou M., Hu Y., Zhang W., Huang J., Gong Y. (2021). ALCAM-EGFR interaction regulates myelomagenesis. Blood Adv..

[23] Chen Y., Huang R., Ding J., Ji D., Song B., Yuan L., Chang H., Chen G. (2015). Multiple myeloma acquires resistance to EGFR inhibitor via induction of pentose phosphate pathway. Sci. Rep..

[24] Raimondo S., Saieva L., Vicario E., Pucci M., Toscani D., Manno M., Raccosta S., Giuliani N., Alessandro R. (2019). Multiple myeloma-derived exosomes are enriched of amphiregulin (AREG) and activate the epidermal growth factor pathway in the bone microenvironment leading to osteoclastogenesis. J. Hematol. Oncol..

[25] Uckun F.M., Qazi S. (2022). ERBB1/EGFR and JAK3 Tyrosine Kinases as Potential Therapeutic Targets in High-Risk Multiple Myeloma. Onco.

[26] Fortschegger K., de Graaf P., Outchkourov N.S., van Schaik F.M.A., Timmers H.T.M., Shiekhattar R. (2010). PHF8 Targets Histone Methylation and RNA Polymerase II To Activate Transcription. Mol. Cell. Biol..

[27] Cruz R.G.B., Madden S.F., Brennan K., Hopkins A.M. (2022). A Transcriptional Link between HER2, JAM-A and FOXA1 in Breast Cancer. Cells.

[28] Hasleton M., Ibbitt J.C., Hurst H.C. (2003). Characterization of the human activator protein-2gamma (AP-2gamma) gene: Control of expression by Sp1/Sp3 in breast tumour cells. Biochem. J..

[29] Liu Q., Borcherding N.C., Shao P., Maina P.K., Zhang W., Qi H.H. (2020). Contribution of synergism between PHF8 and HER2 signalling to breast cancer development and drug resistance. Ebiomedicine.

[30] Vernimmen U., Begon D., Salvador C., Gofflot S., Grooteclaes M., Winkler R. (2003). Identification of HTF (HER2 transcription factor) as an AP-2 (activator protein-2) transcription factor and contribution of the HTF binding site to ERBB2 gene overexpression. Biochem. J..

[31] Nolens G., Pignon J.-C., Koopmansch B., Elmoualij B., Zorzi W., De Pauw E., Winkler R. (2009). Ku proteins interact with activator protein-2 transcription factors and contribute to ERBB2overexpression in breast cancer cell lines. Breast Cancer Res..

[32] Begon D.Y., Delacroix L., Vernimmen D., Jackers P., Winkler R. (2005). Yin Yang 1 Cooperates with Activator Protein 2 to Stimulate ERBB2 Gene Expression in Mammary Cancer Cells. J. Biol. Chem..

[33] Hurst H.C. (2001). Update on HER-2 as a target for cancer therapy: The ERBB2 promoter and its exploitation for cancer treatment. Breast Cancer Res..

[34] Ishii S., Imamoto F., Yamanashi Y., Toyoshima K., Yamamoto T. (1987). Characterization of the promoter region of the human c-erbB-2 protooncogene. Proc. Natl. Acad. Sci. USA.

[35] Das S., Juliana N., Abu Yazit N.A., Azmani S., Abu I.F. (2022). Multiple Myeloma: Challenges Encountered and Future Options for Better Treatment. Int. J. Mol. Sci..

[36] Saldarriaga M.M., Darwiche W., Jayabalan D., Monge J., Rosenbaum C., Pearse R.N., Niesvizky R., Bustoros M. (2022). Advances in the molecular characterization of multiple myeloma and mechanism of therapeutic resistance. Front. Oncol..

[37] Uckun F.M., Qazi S., Demirer T., Champlin R.E. (2018). Contemporary patient-tailored treatment strategies against high risk and relapsed or refractory multiple myeloma. Ebiomedicine.

[38] Gulla’ A., Anderson K.C. (2020). Multiple myeloma: The (r)evolution of current therapy and a glance into future. Haematologica.

[39] Uckun F.M. (2022). Cancer drug resistance in multiple myeloma. Cancer Drug Resist..

[40] Moreau P., Kumar S.K., San Miguel J., Davies F., Zamagni E., Bahlis N., Ludwig H., Mikhael J., Terpos E., Schjesvold F. (2021). Treatment of relapsed and refractory multiple myeloma: Recommendations from the International Myeloma Working Group. Lancet Oncol..

[41] Cavo M., Terpos E., Bargay J., Einsele H., Cavet J., Greil R. (2018). The multiple myeloma treatment landscape: International guide-line recommendations and clinical practice in Europe. Expert Rev. Hematol..

[42] Swamydas M., Murphy E.V., Ignatz-Hoover J.J., Malek E., Driscoll J.J. (2022). Deciphering mechanisms of immune escape to inform immunotherapeutic strategies in multiple myeloma. J. Hematol. Oncol..

[43] Uckun F.M. (2021). Dual Targeting of Multiple Myeloma Stem Cells and Myeloid-Derived Suppressor Cells for Treatment of Chemotherapy-Resistant Multiple Myeloma. Front. Oncol..

[44] Lomas O.C., Tahri S., Ghobrial I.M. (2020). The microenvironment in myeloma. Curr. Opin. Oncol..

[45] Forster S., Radpour R. (2022). Molecular Impact of the Tumor Microenvironment on Multiple Myeloma Dissemination and Extramedullary Disease. Front. Oncol..

[46] Neumeister P., Schulz E., Pansy K., Szmyra M., Deutsch A.J. (2022). Targeting the Microenvironment for Treating Multiple Myeloma. Int. J. Mol. Sci..

[47] García-Ortiz A., Rodríguez-García Y., Encinas J., Maroto-Martín E., Castellano E., Teixidó J., Martínez-López J. (2021). The Role of Tumor Microenvironment in Multiple Myeloma Development and Progression. Cancers.

[48] Giannotta C., Autino F., Massaia M. (2023). The immune suppressive tumor microenvironment in multiple myeloma: The contribution of myeloid-derived suppressor cells. Front. Immunol..

[49] Uckun F.M. (2021). Overcoming the Immunosuppressive Tumor Microenvironment in Multiple Myeloma. Cancers.

[50] Shin S.Y., Lee D.H., Lee J., Choi C., Kim J.-Y., Nam J.-S., Lim Y., Lee Y.H. (2017). C-C motif chemokine receptor 1 (CCR1) is a target of the EGF-AKT-mTOR-STAT3 signaling axis in breast cancer cells. Oncotarget.

[51] Kara I.O., Sahin B., Günesacar R., Unsal C. (2006). Clinical significance of hepatocyte growth factor, platelet-derived growth factor-AB, and transforming growth factor-α in bone marrow and peripheral blood of patients with multiple myeloma. Adv. Ther..

[52] Musolino C., Allegra A., Innao V., Allegra A.G., Pioggia G., Gangemi S. (2017). Inflammatory and Anti-Inflammatory Equilibrium, Proliferative and Antiproliferative Balance: The Role of Cytokines in Multiple Myeloma. Mediat. Inflamm..

[53] Akhtar S., Ali T.A., Faiyaz A., Khan O.S., Raza S.S., Kulinski M., El Omri H., Bhat A.A., Uddin S. (2020). Cytokine-Mediated Dysregulation of Signaling Pathways in the Pathogenesis of Multiple Myeloma. Int. J. Mol. Sci..

[54] Melaccio A., Reale A., Saltarella I., Desantis V., Lamanuzzi A., Cicco S., Frassanito M.A., Vacca A., Ria R. (2022). Pathways of Angiogenic and Inflammatory Cytokines in Multiple Myeloma: Role in Plasma Cell Clonal Expansion and Drug Resistance. J. Clin. Med..

[55] Matsumoto M., Baba A., Yokota T., Nishikawa H., Ohkawa Y., Kayama H., Kallies A., Nutt S.L., Sakaguchi S., Takeda K. (2014). Interleukin-10-Producing Plasmablasts Exert Regulatory Function in Autoimmune Inflammation. Immunity.

[56] Takagi S., Tsukamoto S., Park J., Johnson K.E., Kawano Y., Moschetta M., Liu C.-J., Mishima Y., Kokubun K., Manier S. (2018). Platelets Enhance Multiple Myeloma Progression via IL-1β Upregulation. Clin. Cancer Res..

[57] Narayan P., Osgood C.L., Singh H., Chiu H.-J., Ricks T.K., Chow E.C.Y., Qiu J., Song P., Yu J., Namuswe F. (2021). FDA Approval Summary: Fam-Trastuzumab Deruxtecan-Nxki for the Treatment of Unresectable or Metastatic HER2-Positive Breast Cancer. Clin. Cancer Res..

[58] Kunte S., Abraham J., Montero A.J. (2020). Novel HER2–targeted therapies for HER2–positive metastatic breast cancer. Cancer.

[59] Gupta R., Gupta S., Antonios B., Ghimire B., Jindal V., Deol J., Gaikazian S., Huben M., Anderson J., Stender M. (2022). Therapeutic landscape of advanced HER2-positive breast cancer in 2022. Med. Oncol..

[60] Hideshima T., Anderson K.C. (2021). Signaling Pathway Mediating Myeloma Cell Growth and Survival. Cancers.

[61] Zhou J., Chng W.-J. (2022). Biological Hallmarks and Emerging Strategies to Target STAT3 Signaling in Multiple Myeloma. Cells.

[62] Rana P.S., Soler D.C., Kort J., Driscoll J.J. (2022). Targeting TGF-β signaling in the multiple myeloma microenvironment: Steering CARs and T cells in the right direction. Front. Cell Dev. Biol..

[63] Gilchrist A., Echeverria S.L. (2022). Targeting Chemokine Receptor CCR1 as a Potential Therapeutic Approach for Multiple Myeloma. Front. Endocrinol..

[64] Minakata D., Fujiwara S., Yokoyama D., Noguchi A., Aoe S., Oyama T., Koyama S., Murahashi R., Nakashima H., Hyodo K. (2023). Relapsed and refractory multiple myeloma: A systematic review and network meta-analysis of the efficacy of novel therapies. Br. J. Haematol..

[65] Coira I.F., Rincón R., Cuendet M. (2022). The Multiple Myeloma Landscape: Epigenetics and Non-Coding RNAs. Cancers.

[66] Barreto I.V., Machado C.B., Almeida D.B., Pessoa F.M.C.D.P., Gadelha R.B., Pantoja L.D.C., Oliveira D.D.S., Ribeiro R.M., Lopes G.S., Filho M.O.D.M. (2022). Kinase Inhibition in Multiple Myeloma: Current Scenario and Clinical Perspectives. Pharmaceutics.

[67] Solimando A.G., Malerba E., Leone P., Prete M., Terragna C., Cavo M., Racanelli V. (2022). Drug resistance in multiple myeloma: Soldiers and weapons in the bone marrow niche. Front. Oncol..

[68] Yamamoto L., Amodio N., Gulla A., Anderson K.C. (2021). Harnessing the Immune System Against Multiple Myeloma: Challenges and Opportunities. Front. Oncol..

[69] Minnie S.A., Hill G.R. (2020). Immunotherapy of multiple myeloma. J. Clin. Investig..

[70] Love M.I., Huber W., Anders S. (2014). Moderated estimation of fold change and dispersion for RNA-seq data with DESeq2. Genome Biol..

[71] Von Mering C., Jensen L.J., Snel B., Hooper S.D., Krupp M., Foglierini M., Jouffre N., Huynen M.A., Bork P. (2005). STRING: Known and predicted protein-protein associations, integrated and transferred across organisms. Nucleic Acids Res..

[72] Huber W., von Heydebreck A., Sültmann H., Poustka A., Vingron M. (2002). Variance stabilization applied to microarray data calibration and to the quantification of differential expression. Bioinformatics.

