# Supplementary material for: Upregulated Expression of ERBB2/HER2 in Multiple Myeloma as a Predictor of Poor Survival Outcomes
Source: Int J Mol Sci. 2023 Jun 9;24(12):9943. doi: 10.3390/ijms24129943 (PMC10298070; doi:10.3390/ijms24129943)
Supplement: Supplementary file 1 [file ijms-24-09943-s001.zip › ijms-2412829-supplementary.pdf]

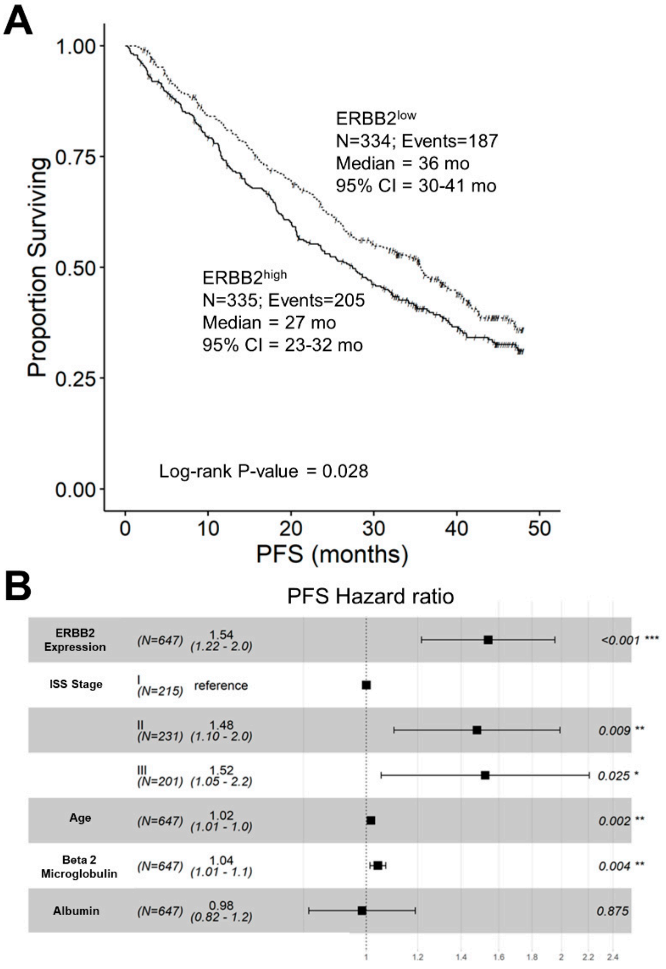

Figure S1. Higher expression levels of ERBB2/HER2 in malignant plasma cells from MM patients is associated with shorter PFS

[**A**] Depicted for comparison are the PFS curves (censored at 48 months) for 335 ERBB2^{high} MM patients (i.e., patients with the top 50% of the highest observed expression level for FPKM-UQ aligned to the ERBB2 sequence) and 334 ERBB2^{low} MM patients (i.e., 334 patients bottom 50% with the lowest observed expression level for FPKM-UQ aligned to the ERBB2 sequence). Cumulative proportion surviving progression-free at 48 mo: 31.1 ± 2.9% for ERBB2^{high} and 35.9 ± 3.1% for ERBB2^{low}. **[B]** Depicted is the Forest plot comparing the HRs for each covariate included in a multivariate Cox proportional hazards model, namely ERBB2 mRNA level, ISS stage, age, as well as serum albumin and beta 2 microglobulin levels. ERBB2 mRNA expression level exhibited a significant 1.54-fold increase in the hazard ratio for each unit increase in FPKM-UQ counts (P < 0.001). Significant p-values are indicated by *, ** and *** for p < 0.05, p < 0.01 and p < 0.001 respectively.

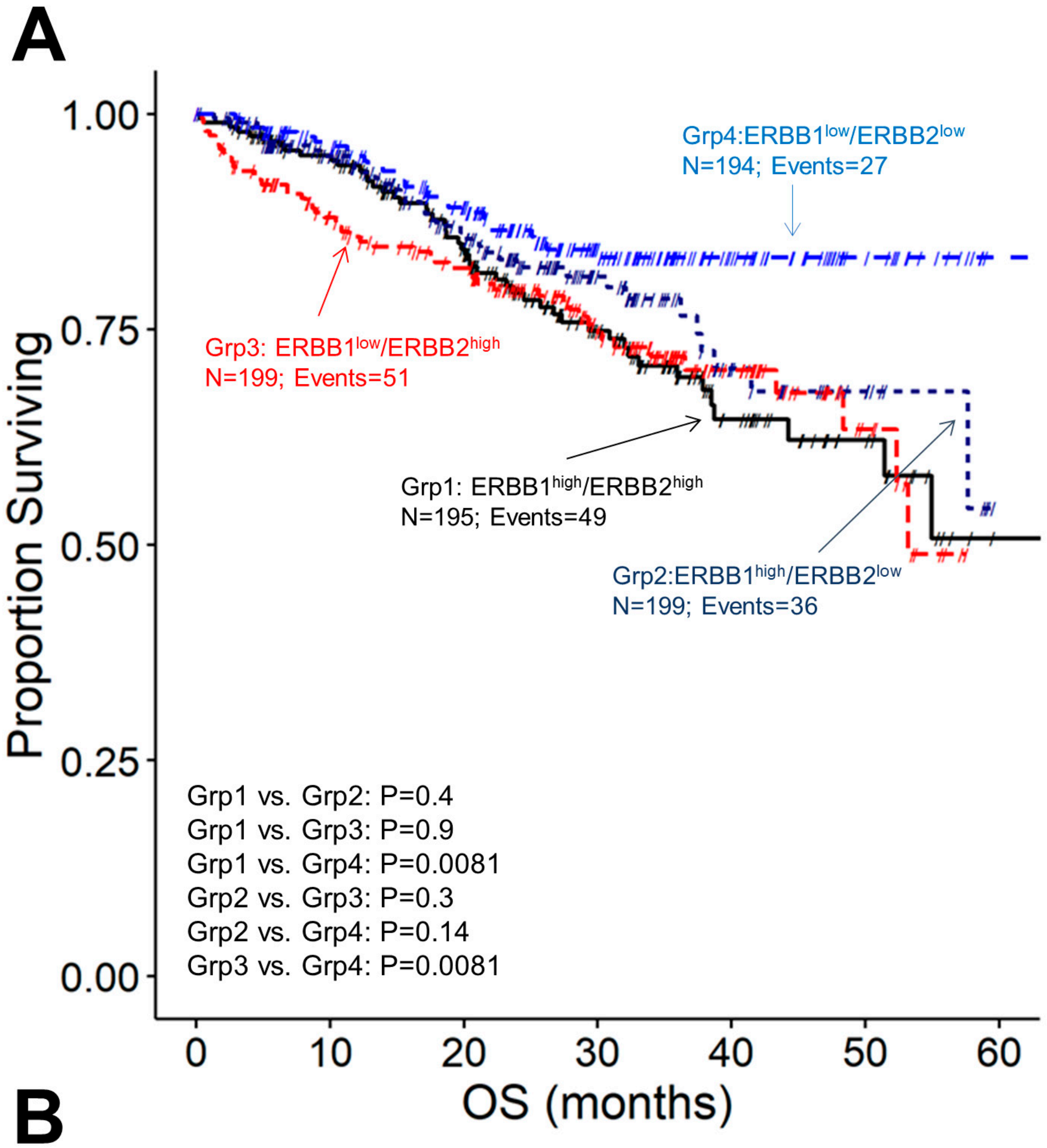

B

Group	ERBB1/ERBB2 expression	N	#Events	Median	Lower 95% CI	Upper 95% CI	Cumulative surviving at 60 months (%)
Grp1	$ERBB1^{high}/ERBB2^{high}$	195	49	>60 mo	51.4	NA	50.8 ± 9
Grp2	$ERBB1^{high}/ERBB2^{low}$	199	36	>60 mo	57.7	NA	54.3 ± 12.9
Grp3	$ERBB1^{low}/ERBB2^{high}$	199	51	53.2 mo	52.3	NA	48.9 ± 10
Grp4	$ERBB1^{low}/ERBB2^{low}$	194	27	>60 mo	NA	NA	83.3 ± 3

Figure S2. The prognostic impact of ERBB1 and ERBB2 on Overall Survival (OS) in newly diagnosed MM patients from the MMRF-CoMMpass study. [**A**] Depicted for comparison are the OS curves for 4 distinct patient groups defined by co-expression levels of ERBB2 and ERBB1: (i)

ERBB1^{high}/ERBB2^{high} (top 50th percentile of the highest observed expression levels for both ERBB1 and ERBB2 mRNA expression levels) (N=195), (ii) ERBB1^{low}/ERBB2^{low} (bottom 50th percentile of the lowest observed expression levels for both ERBB1 and ERBB2 (N=194), (iii) ERBB1^{low}/ERBB2^{high} (N=199) and (iv) ERBB1^{high}/ERBB2^{low}(N=199). [**B**] ERBB1^{low}/ERBB2^{low} patients (Grp4) had fewer deaths and the best 5-year survival of 83.3%.

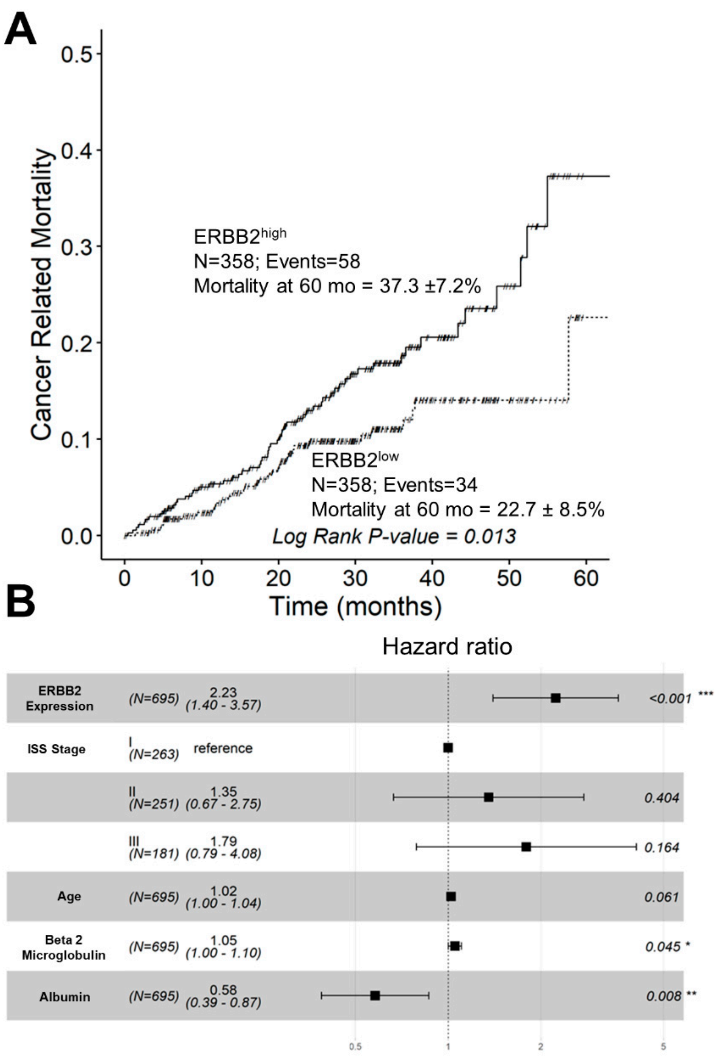

Figure S3. Amplified expression of ERBB2 is associated with increased cancer-related mortality (CRM) in newly diagnosed MM patients from the MMRF-CoMMpass study.

[**A**] Depicted for comparison are the CRM curves (censored at 60 months) for 358 $ERBB2^{high}$ patients (i.e., patients with the top 50% of the highest observed expression level for FPKM-UQ aligned to the ERBB2 sequence) and 358 $ERBB2^{low}$ patients (i.e., patients bottom 50% with the lowest observed expression level for FPKM-UQ aligned to the ERBB2 sequence). [**B**] Depicted is the Forest plot comparing the HRs for each covariate included in a multivariate Cox proportional hazards model, namely ERBB2 mRNA level, ISS stage, age, as well as serum albumin and beta 2 microglobulin levels. ERBB2 mRNA expression level exhibited a significant 2.23-fold increase in the hazard ratio for each unit increase in FPKM-UQ counts (P < 0.001). Significant P-values are indicated by *, ** and *** for p < 0.05, p < 0.01 and p < 0.001 respectively.

A

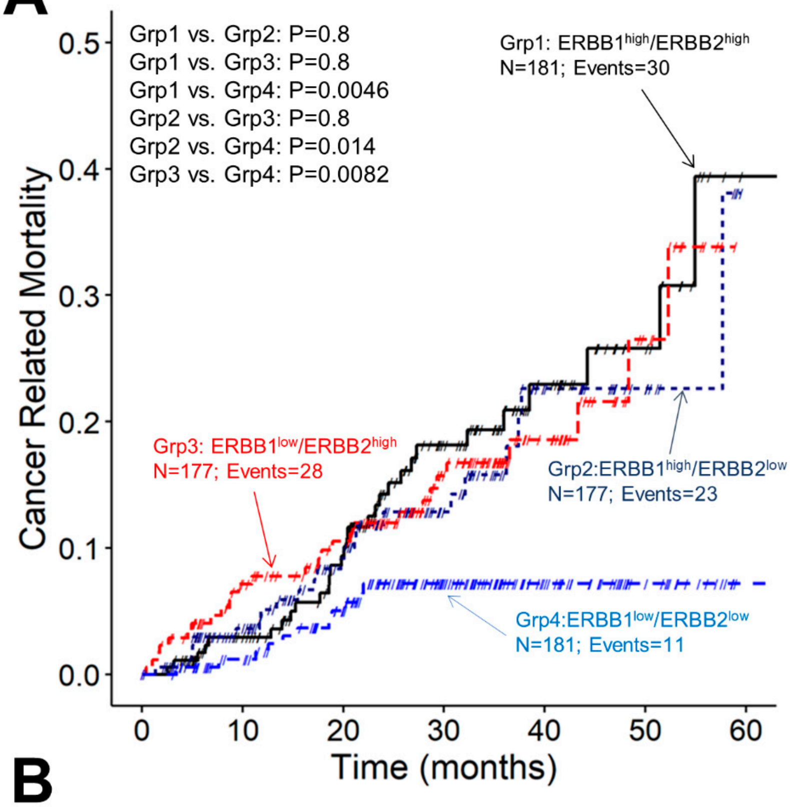

B

Group	ERBB1/ERBB2 expression	N	#Events	Median	Lower 95% CI	Upper 95% CI	Cancer Related Mortality at 60 months (%)
Grp1	$ERBB1^{high}/ERBB2^{high}$	181	30	>60 mo	54.9	NA	39.4 ± 10
Grp2	$ERBB1^{high}/ERBB2^{low}$	177	23	>60 mo	57.7	NA	38.1 ± 14.4
Grp3	$ERBB1^{low}/ERBB2^{high}$	177	28	>60 mo	NA	NA	33.8 ± 9
Grp4	$ERBB1^{low}/ERBB2^{low}$	181	11	>60 mo	NA	NA	7.2 ± 2.1

Figure S4. The prognostic impact of ERBB1 and ERBB2 on cancer-related mortality (CRM) in newly diagnosed MM patients from the MMRF-CoMMpass study.

[**A**] Depicted for comparison are the CRM curves for 4 distinct patient groups defined by co-expression levels of ERBB2 and ERBB1: (i) $ERBB1^{high}/ERBB2^{high}$ (top 50th percentile of the highest observed expression levels for both ERBB1 and ERBB2 mRNA expression levels) (N=181), (ii) $ERBB1^{low}/ERBB2^{low}$ (bottom 50th percentile of the lowest observed expression levels for both ERBB1 and ERBB2 (N=181) , (iii) $ERBB1^{low}/ERBB2^{high}$ (N=177) and (iv) $ERBB1^{high}/ERBB2^{low}$(N=177). [**B**] $ERBB1^{low}/ERBB2^{low}$ patients representing 25.3% of the 716 evaluable patients had significantly fewer cancer-related deaths consequently the lowest 5-year CRM (7.2±2.1%) when compared to the remaining 3 groups of patients (5-year CRM: $ERBB1^{high}/ERBB2^{high}$ = 39.4 ± 10%, $ERBB1^{high}/ERBB2^{low}$ = 38.1 ± 14.4%, $ERBB1^{low}/ERBB2^{high}$ = 33.8 ± 9%).
